# Aligned hierarchical Ag/ZnO nano-heterostructure arrays via electrohydrodynamic nanowire template for enhanced gas-sensing properties

**DOI:** 10.1038/s41598-017-12553-7

**Published:** 2017-09-22

**Authors:** Zhouping Yin, Xiaomei Wang, Fazhe Sun, Xiaohu Tong, Chen Zhu, Qiying Lv, Dong Ye, Shuai Wang, Wei Luo, YongAn Huang

**Affiliations:** 1 State Key Laboratory of Digital Manufacturing Equipment and Technology, Huazhong University of Science and Technology, Wuhan, 430074 China; 2 Flexible Electronics Research Center, Huazhong University of Science and Technology, Wuhan, 430074 China; 3 School of Science, Shandong University of Technology, Zibo, 255100 China; 4 Analysis Testing Center, Shandong University of Technology, Zibo, 255100 China; 5 School of Optical and Electronic Information, Huazhong University of Science and Technology, Wuhan, 430074 China; 6 School of Chemistry and Chemical Engineering, Huazhong University of Science and Technology, Wuhan, 430074 China

## Abstract

Gas sensing performance can be improved significantly by the increase in both the effective gas exposure area and the surface reactivitiy of ZnO nanorods. Here, we propose aligned hierarchical Ag/ZnO nano-heterostructure arrays (h-Ag/ZnO-NAs) via electrohydrodynamic nanowire template, together with a subsequent hydrothermal synthesis and photoreduction reaction. The h-Ag/ZnO-NAs scatter at top for higher specific surface areas with the air, simultaneously contact at root for the electrical conduction. Besides, the ZnO nanorods are uniformly coated with dispersed Ag nanoparticles, resulting in a tremendous enhancement of the surface reactivity. Compared with pure ZnO, such h-Ag/ZnO-NAs exhibit lower electrical resistance and faster responses. Moreover, they demonstrate enhanced NO_2_ gas sensing properties. Self-assembly via electrohydrodynamic nanowire template paves a new way for the preparation of high performance gas sensors.

## Introduction

The quantitative detection of noxious gas such as Nitrogen dioxide (NO_2_) has been drawn considerable attention. Semiconducting metal oxides, especially ZnO, have been commonly used as the active sensing materials for gas sensors due to their fast response, short response-recovery time, excellent electrical performance, and long term stability^1–3^. Recently, researches have focused on enhancing the gas sensing performance of ZnO nanostructures by using nanostructured materials with ultra-high specific surface areas^4–6^, appropriate element doping^7–10^, surface decoration with noble metals (Au, Pt, Pd, and Ag)^11–14^ nanocomposite^15–17^ and construction of heterostructure^18–22^. Among them, the hierarchical nano-heterostructures have attracted great attention due to their rich architectures, extraordinary properties, and novel applications^23,24^. The decoration of nanostructures with noble metal nanoparticles can further enhance multiple gas-sensing performance^25^. A simple way was developed to fix Au on ZnO microrods, and the obtained Au decorated ZnO microrods showed enhanced sensing performance towards ethanol detection^26^. The preparation of Pt modified ZnO nanowires was demonstrated for room temperature ethanol sensing^27^. ZnO nanostructures decorated with Pd were synthesized through self-assemblies, and the obtained hybrid material exhibited a great improvement on sensitivity to H_2_S gas^28^. Unfortunately, most ZnO film-like precursor are too dense so that the ZnO nanorods usually exhibit rather compact. The exposure area of the target gas molecules in sensing is too low even if the ZnO nanorods modified with noble metals or oxides. A high exposure area with the gas can be achieved at the case of low-density ZnO nanorods; however, the conductivity is usually below the minimum detection limit.

It is important to address the paradox between the conductivity of the cluster of ZnO nanostructures and the exposure area with the gas. When ribbon-like precursor are adopted, ZnO nanorods at edges and corners would scatter to enhance the specific area, while the others still keep similar with those grown on film-like precursor. By replacing ribbon-like precursor with nanowire-like one, the specific area of ZnO nanorods will be futher improved as a result of the formation of brush-like hierarchical nanostructures, and the tight contact of nanorods at root is also beneficial to the transfer of electrons. However, the traditional synthesis methods^29^, such as traditional ink-jet printing, aerosol-based nanotechnology, physical vapor deposition, chemical vapor deposition and hydrothermal method, are unable to fabricate surface-modified, aligned hierarchical nanostructures.

In view of this, herein, we present a newly-proposed approach to fabricate aligned hierarchical Ag/ZnO nano-heterostructure arrays (Ag/ZnO-NAs) in a digital, large-area and cost-effective manner, through electrohydrodynamic direct-writing polymer nanowire template followed by hydrothermal growth and photoreduction. The Ag/ZnO-NAs combine the advantage of surface modification and hierarchical nano-heterostructures, and this effect tremendously enhance the gas sensing performances. Moreover, the Ag/ZnO-NAs exhibit an excellent selectivity for NO_2_ gas sensing. The gas sensing performance of hierarchical ZnO nanorods with various precursor pattern (line-width and gap) and Ag contents (corresponding to Ag+ photoreduction time) has been systematically evaluated and the optimal layout of precursor and photoreduction time have been discovered. The corresponding mechanism has been discussed as well.

## Results and Discussion

**Figure 1:**
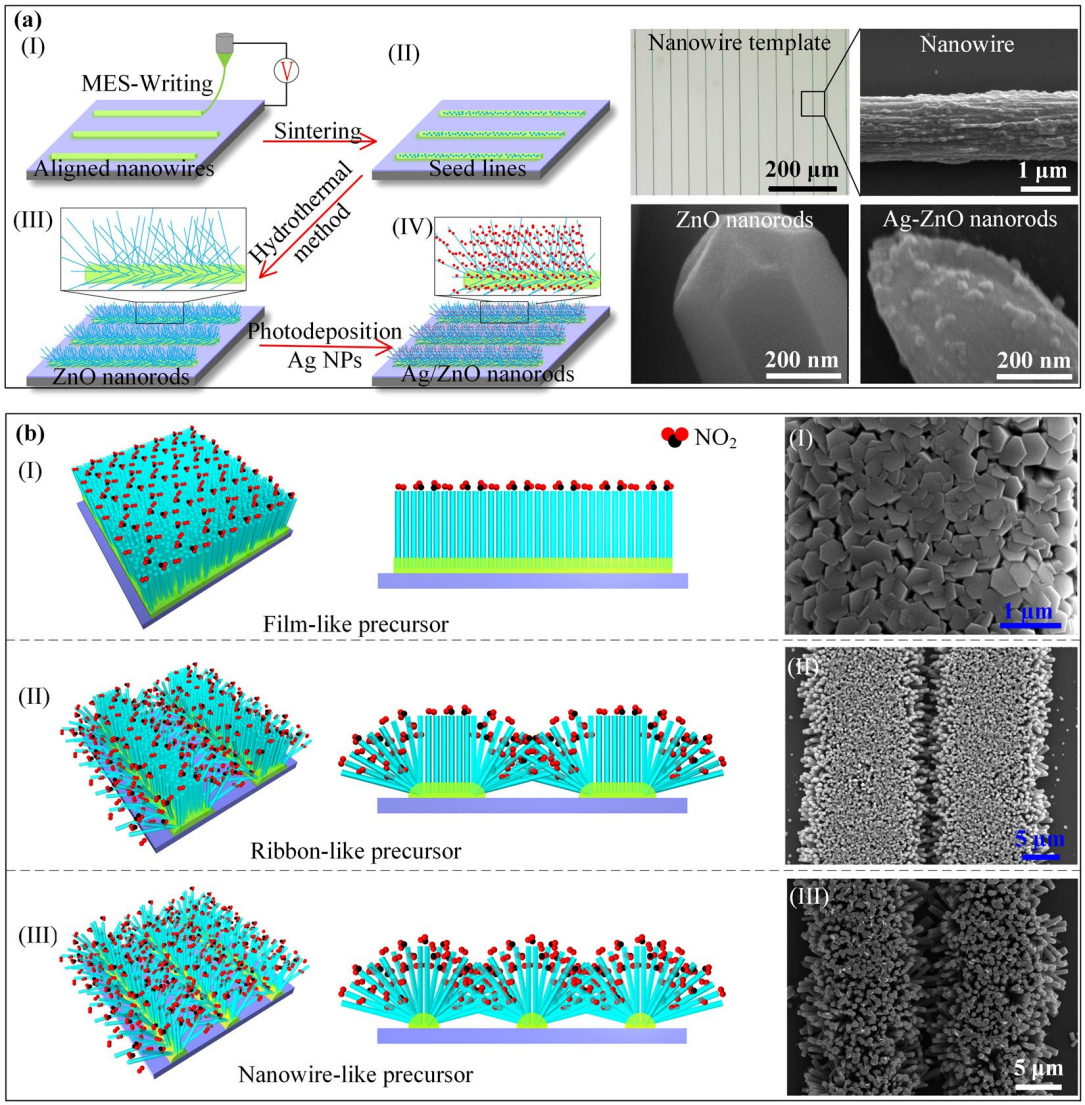
(**a**) The process steps of fabrication of Ag-ZnO NAs, and the optical images of nanowire template, FESEM image of nanowire, and FESEM images of ZnO nanorods and Ag-ZnO nanorods. (**b**) The schematic oblique and sectional views of NO_2_ absorption of ZnO nanorod arrays from (I) film-like, (II) ribbon-like, and (III) nanowire-like precursors, respectively, and FESEM images of various ZnO-NAs grown on film-like, ribbon-like and nanowire-like seed layers, respectively.

Figure 1a conceptually depicts the stepwise manufacturing procedure brush-like Ag/ZnO-NAs on a nanowire-like precursor by digital mechanoelectrospinning direct-writing (MES-Writing) technology^29,30^, together with a subsequent hydrothermal synthesis and photoreduction reaction. Firstly, the digital MES-Writing technology was adopted to deposited large-scale aligned electrohydrodynamic nanowire template with high-density ZnO seed. The template can be fabricated in large-area substrate, and the diameter of nanowires is from 200 nm to 20 μm. The nanowire-diameter and nanowire-gap could be controlled digitally with arbitrary value. Secondly, these samples were put into oven with 200 °C for 2 hours, to form ZnAc/PEO nanoscale crystal nucleus. Thirdly, the hydrothermal synthesis method was utilized to selectively grow ZnO-NAs on the printed precursor pattern. The sample exhibits aligned parallel array and brush-like morphology of ZnO-NAs. The density of ZnO nanorods is determined by the seeds, the shape of ZnO nanorods results from the growth time of hydrothermal method, and the layout of ZnO nanorods depends on the aligned nanowires. It can be seen that uniform nanorods with diameters of ~400 nm and length of dozens of micrometers have been fabricated. Finally, Ag nanoparticles (Ag-NPs) are photoreduced on the surfaces of ZnO nanorods to form Ag/ZnO-NAs. It is worth mentioning that the morphology of the nanorod arrays remain the same even if the surface of ZnO nanorods are grown of Ag nanoparticles. The high-magnification field emission scanning electron microscopy (FESEM) images illustrate that the Ag-NPs with less than 50 nm in size are randomly and uniformly distributed on the surface of ZnO nanorods.

Figure 1b schematically and experimentally illustrates I) the compact ZnO nanorod arrays (ZnO-NAs) growing on film-like precursor, II) hybrid ZnO-NAs on ribbon-like precursor, and III) brush-like ZnO-NAs on nanowire-like precursor, from oblique view, front view and top view, respectively. For comparison, the ZnO NAs grown on film-like and ribbon-like precursors, respectively, are prepared with the same procedure. As shown in Fig. 1b (I and II), the ZnO nanorods on film-like precursor and the middle part of ribbon-like precursor crowd together, while the ZnO nanorods at edges of ribbon are interdigitated between adjacent parallel arrays^31^. The brush-like 3D h-Ag/ZnO-NAs compose of uniform nanoparticles, vertical nanorods and aligned nanowires. Obviously, the resolution and gap of deposited lines determine the exposure area with target gas molecules and reduced Ag nanoparticles (Ag-NPs). Therefore, more area of the brush-like ZnO-NAs on nanowire-like precursor can be exposed to target gas molecules so that the change in conductance of brush-like nanostructures will be greater. Figure 1b (III) shows the samples exhibit aligned parallel arrays and brush-like morphology of ZnO-NAs. The brush-like morphology contributes to Ag-NPs grown controllably on the surface of ZnO nanorods, and is chosen to form h-Ag/ZnO-NAs of nanoparticles(Ag)-on-nanorods(ZnO)-on-nanowires(precursor). Meanwhile high-density ZnO-NAs prevent Ag-NPs from growing on the surface of ZnO nanorods, and prevent the Ag/ZnO nanorods from contacting with gas. When brush-like Ag/ZnO-NAs are exposed to target gas, the change of conductance will be greater.

### Morphologies and Structures

**Figure 2:**
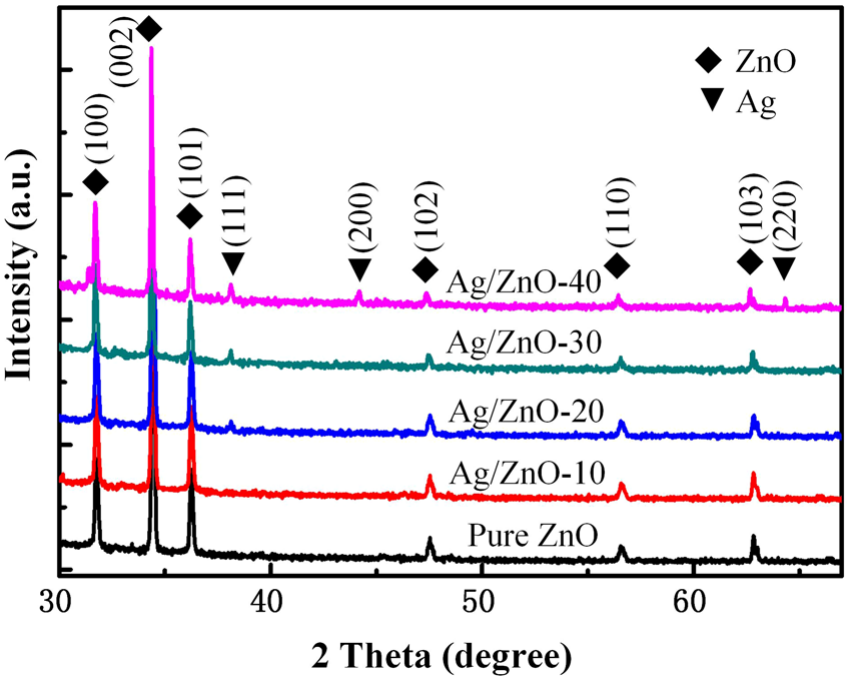
The XRD of samples of pure ZnO, Ag/ZnO-10, Ag/ZnO-20, Ag/ZnO-30 and Ag/ZnO-40.

Structure and component of Ag/ZnO-NAs samples were confirmed by X-ray diffraction (XRD) and Transmission electron microscope (TEM). Typical XRD analysis of various Ag/ZnO samples are shown in Fig. 2, where samples photoreduced for 10 min, 20 min, 30 min, 40 min are marked as Ag/ZnO-10, Ag/ZnO-20, Ag/ZnO-30 and Ag/ZnO-40, respectively. The recorded diffraction patterns can be indexed into two groups: ZnO crystal and Ag^+^. There are six well defined diffraction peaks (marked as “♦”) at 2θ = 31.88°, 34.41°, 36.26°, 47.52°, 56.56° and 62.9° in all diffraction patterns, which correspond to (100), (002), (101), (102), (110) and (103) planes of ZnO crystal. This is the typical hexagonal wurtzite phase of ZnO. The lattice constants of ZnO are a = 3.2498 Å and c = 5.2066 Å (JCPDS card no. 36–1451). (002) peak has the maximum intensity, indicating that c-axis [0001] is the growth direction of ZnO nanorods. The other observed characteristic diffraction peaks of 38.2°, 44.4° and 64.6° (marked as “▼”) match well with the (111), (200) and (220) peaks of Ag with the face-centered cubic (FCC) structure (JCPDS card no. 04–0783) with the lattice constant of a = 4.0862 Å, which proves that Ag^+^ is photoreduced successfully. Additionally, there exist no characteristic peaks corresponding to other impurities, e.g. AgO and Ag_2_O etc.

The XRD analysis shows the successful preparation of a binary phase consisting of wurtzite ZnO and FCC Ag in the samples. In addition, every ZnO diffraction peak has no notable shift for ZnO/Ag composites compared with pure ZnO samples. It indicates well crystal structure of the as-synthesized Ag/ZnO-NAs that Ag neither changes the intrinsic properties of ZnO nanocrystal nor incorporates into the lattice of ZnO. The expansion or shrinkage of ZnO lattice for Ag/ZnO nanocrystals should be negligible. By comparing peaks of ZnO and Ag, it is found that the peaks intensity of ZnO are gradually decreased and that of Ag nanoparticle are evidently increased evidently, with increasing the photoreduction time of Ag^+^.

**Figure 3:**
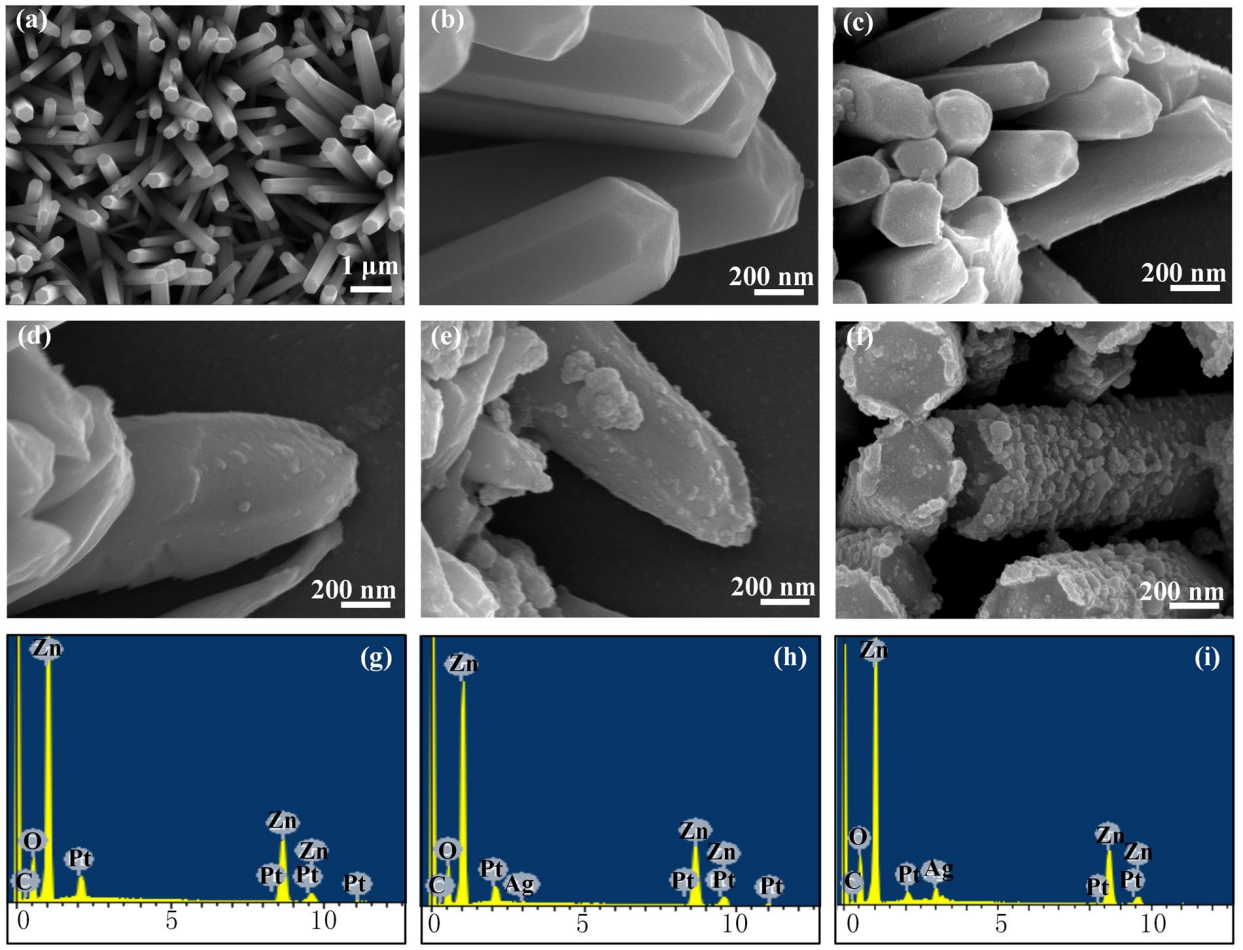
The FESEM images of (**a**,**b**) pure ZnO nanorods, (**c**) Ag/ZnO-10, (**d**) Ag/ZnO-20, (**e**) Ag/ZnO-30 and (**f**) Ag/ZnO-40. The EDXS spectra of (**g**) pure ZnO, (**h**) Ag/ZnO-30 and (**i**) Ag/ZnO-40.

Figure 3 shows the FESEM images and energy dispersive X-ray spectrum (EDXS) spectrums of the obtained samples. The density of Ag-NPs on ZnO nanorods is determined by the photoreduction time, and the large cover area benefits from the specific area of brush-like structures. The comparison between ZnO nanorods and Ag-ZnO nanorods can be observed from the FESEM images. The size of Ag-NPs keeps almost constant when the photoreduction time is less than 30 min, but sharply increases when the photoreduction time reaches 40 min, and some of Ag-NPs aggregate into agglomerates (Fig. 3f). The gap between nanowires plays another critical role in the improvement of specific area. The length of ZnO nanorods determines the optimal gap between lines, and the optimal gap is ~20 μm. The morphologies of the Ag/ZnO nanorods with various Ag content are shown in Fig. 3(c,d,e,f). The surfaces of Ag/ZnO nanorods are rough compared to that of pure ZnO nanorods. Additionally, the quantity of the Ag-NPs monotonously increases with the photoreduction time, which agrees with XRD analysis of Fig. 2.

The Ag-NPs can grow uniformly on both top and lateral surfaces of ZnO nanorods, which is attributed to several reasons. Free electron, Ag^+^ and UV light are the necessary conditions for the formation of Ag-NPs. When the surface of ZnO-NAs are irradiated with UV light (400 W) many electrons will be accumulated on both top and lateral surfaces of ZnO nanorods. The brush-like ZnO-NAs, on one hand, is favorable for the diffusion of Ag^+^ on the surfaces of ZnO nanorods. On the other hand, it plays a role as reflector in light trapping structure, which reflects the UV light to the root of ZnO nanorod. Thus, Ag-NPs can be easily formed on both top and lateral surface of ZnO nanorods.

**Figure 4:**
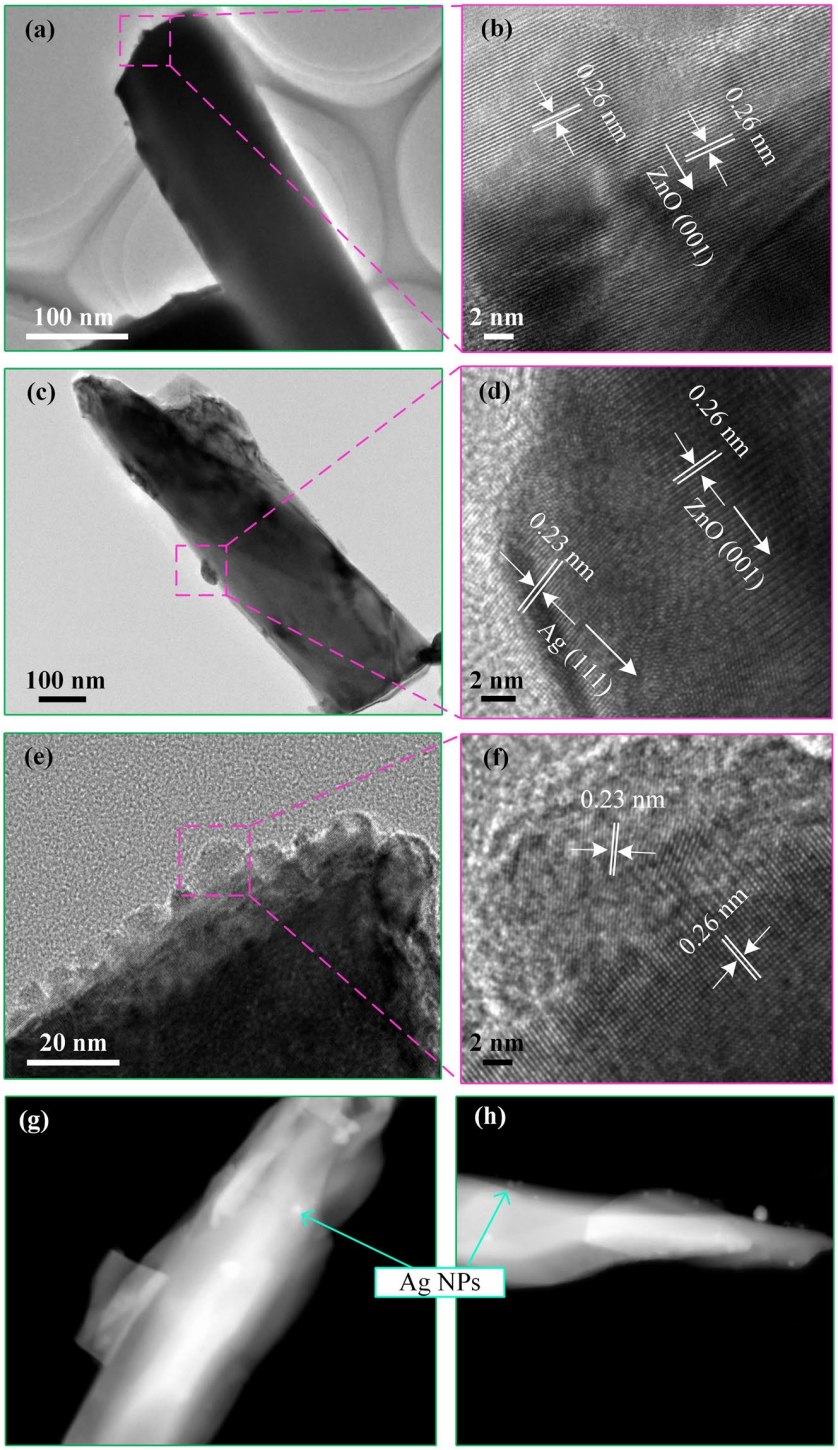
TEM images of (**a**,**b**) pure ZnO and (**c**,**d**) Ag/ZnO-30, (**e**,**f**) Ag/ZnO-40 nanostructures, and HAADF-STEM images of (**g**) Ag/ZnO-30 and (**h**) Ag/ZnO-40.

TEM/HAADF-STEM/HRTEM images further reveal the structure of Ag/ZnO samples. As shown in Fig. 4c–f, the TEM images show that Ag/ZnO-30 and Ag/ZnO-40 are covered with dispersed Ag-NPs (highlighted by red dash-line quadrangles) with an average diameter of dozens of nanometers in size, while there is no attachment for pure ZnO nanorods (Fig. 4a,b). EDXS image shows that except the C and Pt elements arise from sprayed carbon and Pt before scanning, the sample only contains the element of Zn, O, and Ag. In addition, the Ag peak intensity of Ag/ZnO-40 (Fig. 3i) is higher than that of Ag/ZnO-30 (Fig. 3h). It indicates that Ag-NPs grow well on the surface of ZnO nanorods. There are no other element peaks, which is consistent with the analysis result of XRD. The bright spots in cyan solid line circles in the HAADF-STEM images reveal that the Ag-NPs are distributed along the ZnO nanorods (Fig. 4g and h). The quantity of Ag-NPs in Fig. 4e is larger than that in Fig. 4c due to the increase of Ag^+^ photoreduction time. HRTEM images (Fig. 4b,d and f) show that the lattice fringes of ZnO and Ag. The lattice fringes of pure ZnO nanorods are revealed clearly in Fig. 4b. The measured lattice space is 0.26 nm, which is the space of the (001) plane, revealing that the growth direction of ZnO nanorods is [0001]. The lattice fringes with inter planar spacing of 0.23 nm are corresponding to the (111) plane of FCC Ag (Fig. 4d,f). So ZnO-NAs are successfully modified by Ag NPs, which is in good agreement with the results of XRD.

### Sensing mechanism

The main carriers are free electrons of the conduction band (C.B.) for n-type ZnO, and the main charge acceptor on ZnO surface is oxygen molecule. Thus the free electrons in C.B. of ZnO ionized the adsorbed oxygen species, and ionized oxygen species such as O_2_^−^, O^−^ and O^2−^ were formed^32^. Oxygen adsorption reactions highly depend on working temperature, and the stable oxygen ions are O_2_^−^, O^−^ and O^2−^ at below 100 °C, within 100–300 °C and above 300 °C operating temperature, respectively^33^. The lower concentration of free electrons in C.B. of ZnO increases the resistance^34^. When NO_2_ gas molecules are adsorbed on the surface of ZnO nanorods, it attracts the free electrons from the C.B. of ZnO. NO_2_ molecules have higher electrophilic property, and they can capture the electronics from ZnO and react with the oxygen ions to decrease the electron concentration. The surface depletion region of ZnO nanorods is further widen, so the resistance further increases. All the involved reactions are described in detail in Supporting Information. When NO_2_ concentration increases, more electrons will be involved in above reactions. The resistance of ZnO samples is modulated by the adsorption and desorption of gas molecules.

**Figure 5:**
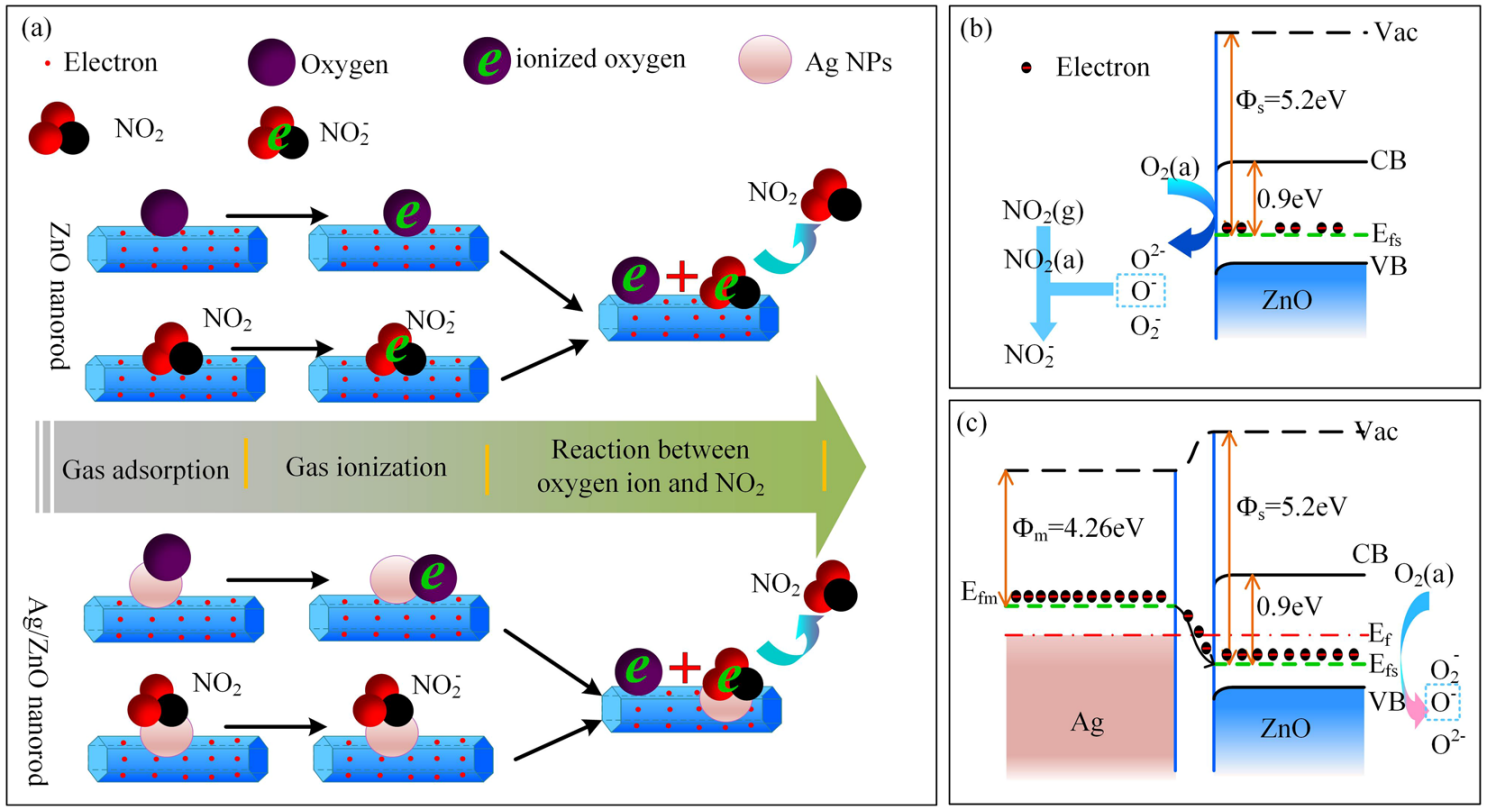
The chemical sensitization mechanism (**a**) of catalytic property for Ag-NPs on ZnO nanorods. The energy band structures of pure ZnO (**b**) and the Fermi energy level equilibrium of Ag-ZnO junction (**c**).

The Ag-NPs is attribute to gas sensing performance based on catalytic surface reactivity of Ag-NPs and the Schottky junctions formed on the interface between Ag and ZnO^35^. Figure 5a shows the schematic diagram of catalytic property for Ag/ZnO sensor. The Ag-NPs can tune carrier concentration in ZnO nanorods. The oxygen molecule is adsorbed on Ag catalyst preferentially and then spill over to ZnO matrix. The NO_2_ molecules are adsorbed onto the surface of Ag-NPs and then react with oxygen species and electrons. The electrons captured by adsorbed oxygen ions are delivered, leading to an enhanced sensing response. The effect of Schottky junction refers to the electron exchange between Ag and ZnO, enhancing the separation of charge carriers and lifetime of electrons^36^. For pure ZnO sensor, the absorbed oxygen is ionized via combining with the spill-over electrons of the Fermi energy level (*E*_*fs*_) in ZnO (Fig. 5b). The work function (*Ф*_*s*_) of ZnO (5.2 eV) is larger than that of silver (*Ф*_*m*_ = 4.26 eV), and the *E*_*fs*_ of ZnO is lower than the Fermi energy level of Ag (*E*_*fm*_). When Ag-NPs are adopted, partial electrons migrate from Ag to ZnO until the two systems attain equilibrium and the new uniform Fermi energy level is formed (*E*_*f*_), as described in Fig. 5c. When *E*_*f*_ > *E*_*fs*_, more oxygen will be absorbed. Above two mechanisms both could modulate the sensing reactions between target gas and oxygen species to realize better sensing performance.

### The effect of Ag-NPs on gas sensing performance of ZnO-NAs

**Figure 6:**
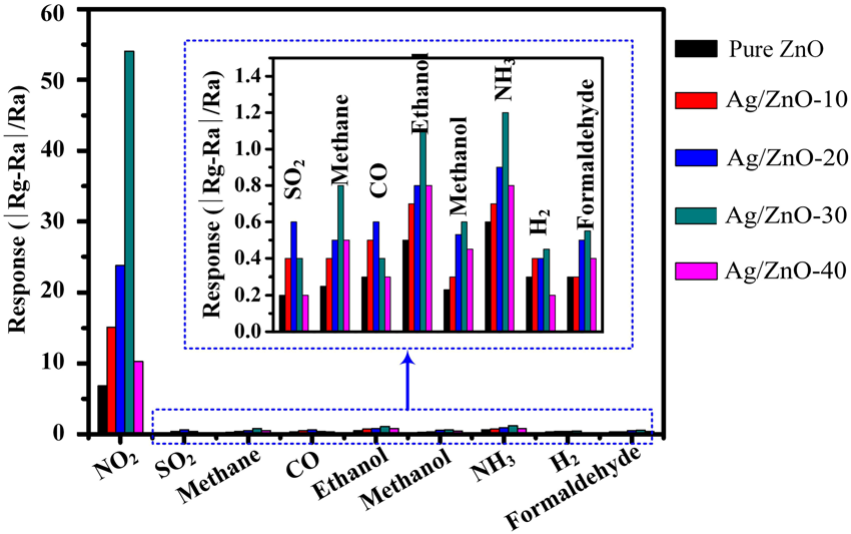
Responses of pure ZnO and Ag-ZnO samples toward NO_2_(10 ppm), SO_2_(50 ppm), methane (50 ppm), CO(10 ppm), ethanol(10 ppm), methanol(10 ppm), NH_3_(50 ppm), H_2_(10 ppm), and formaldehyde (10 ppm) at the working temperature of 225 °C.

The chemical sensing performances of these samples were then investigated. The resistance (*R*) of these samples was tested in clean and dry air to get the baseline resistance *Ra*. *R* increases (decreases) to a maximum (minimum) value *Rg* when the sensor is placed in different concentrations of target gas. Sensor response *S* is defined as *S* = (*Rg*−*Ra*)/*Ra* for oxidizing gas and *S* = (*Ra*−*Rg*)/*Ra* for reducing gas. The responses of ZnO and Ag/ZnO samples to different kinds of gases are shown in Fig. 6. The Ag/ZnO-based sensors show much higher response to NO_2_ gases than other gases such as SO_2_, methane, CO, ethanol, methanol, NH_3_, H_2_, and formaldehyde at the working temperature of 225 °C. The highest response is 54.0 to 10 ppm NO_2_ but is less than 1.2 toward other gases even at higher concentration. So the samples show highly selective sensing properties for NO_2_ gas, which can distinguish the NO_2_ gas among the mixture gases. It is consistent with the results obtained by other authors^37^.

**Figure 7:**
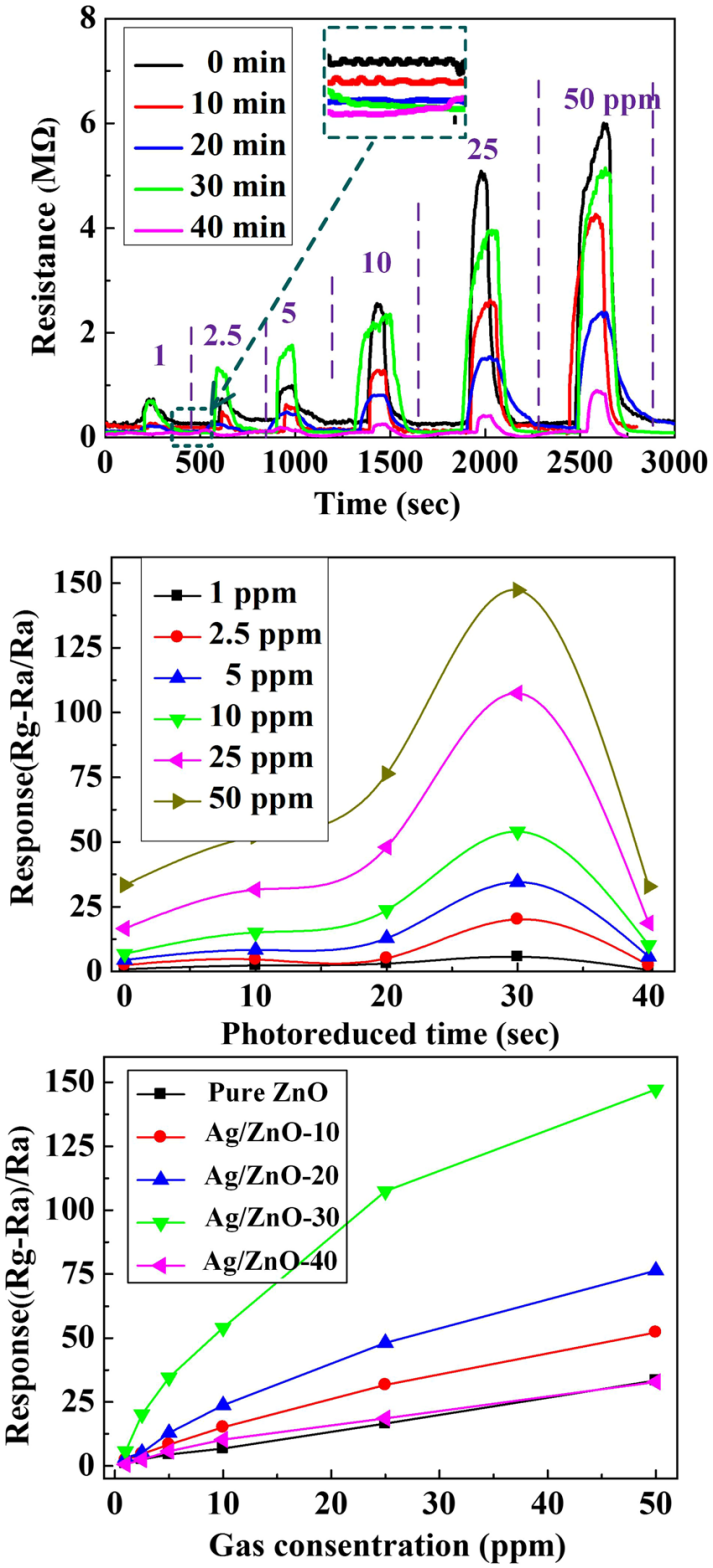
(**a**) The dynamic responses of pure ZnO and Ag-ZnO samples in different NO_2_ concentrations. The inset of figure (**a**) is the baseline of samples. Response (**b**,**c**) of the pure ZnO and Ag-ZnO sensors to different concentrations of NO_2_ at the same working temperature.

Figure 7a shows dynamic responses of ZnO and Ag/ZnO samples to NO_2_ at various concentrations (from 1 to 50 ppm) at 225 °C. Ag/ZnO samples are n-type gas sensors like pure ZnO, whose resistances increase to maximum value after injection of oxidizing NO_2_ gas into the testing chamber. The response to NO_2_ is rapid, steady and reproducible when the samples are in NO_2_ atmosphere. The responses of pure ZnO and Ag/ZnO sensors both increase with NO_2_ concentration increasing. The resistances can recover to the original baseline level completely with removing NO_2_ for all samples. The morphologies of ZnO-NAs have large advantage for gas sensing. The brush-like morphology of ZnO-NAs is more beneficial to gas transmission and adsorption than that of vertically compact ZnO-NAs^38^. the interdigitated nanorods between the adjacent arrays contribute to gas response. Moreover, most junctions between nanorods are point junctions rather than cross junctions and block junctions, which is beneficial for gas sensing performance^39^. ZnO nanorods are oriented in [0001], which have the best gas sensing activity^40^. Because the [0001] plane terminated with Zn^2+^ ion is concerned, which is able to seize atmosphere O_2_ through physical/chemical absorption.

Ag-NPs release free electrons which increase carrier concentration and conductance of ZnO. So the baseline resistance *Ra* of sensors is decreased when the photoreduction time of Ag^+^ ions increase (the inset of Fig. 7a). The *Ra* of pure ZnO, Ag/ZnO-10, Ag/ZnO-20, Ag/ZnO-30 and Ag/ZnO-40 are 0.17 MΩ, 0.1 MΩ, 0.04 MΩ, 0.035 MΩ and 0.016 MΩ, respectively. Gas response increases with the photoreduction time (the Ag content) up to 30 min and then decreases sharply for Ag/ZnO-40 sample (Fig. 7b). It indicates that NO_2_ sensing property of ZnO nanorods sensor can be enhanced by Ag-NPs modification, and reach the highest response value at the photoreduction time of 30 min. When Ag-NPs exceed the optimal content, they reversely behave as recombination centers of charge carriers^41^, which is caused by the electrostatic attraction of charged Ag (negatively) and charged holes (positively)^42^. When the concentrations of NO_2_ are 1 ppm, 2.5 ppm, 5 ppm, 10 ppm, 25 ppm, 50 ppm, the responses of pure ZnO sample are 0.96, 2.56, 4.47, 6.84, 16.59, 33.44, and the responses of Ag/ZnO-30 are 5.77, 20.16, 34.53, 54.07, 107.43, 147.19, respectively. The responses of Ag/ZnO-30 are at least two times higher than that of pure ZnO-NAs for the same concentration of NO_2_. Meanwhile, the response time (less than 120 s) and recovery time (less than 150 s) for all h-Ag/ZnO-NAs gas sensor at every test concentrations of NO_2_ are of the order of mins. And the detection limits can be achieved 1 ppm or less.

In addition, gas sensing performance can be described by the tangents of response curves. The tangent of Ag/ZnO-30 sample response curve (Fig. 7c) is larger than that of other samples at various gas concentrations. Gas response decreases sharply for Ag/ZnO-40 sensors, since too much surface of ZnO is occupied by Ag-NPs, which influences the adsorption of oxygen and NO_2_ gas molecular. So Ag/ZnO-30 gas sensor has the best sensing property. It indicates that the gas responses increase when NO_2_ concentration increases (Fig. 7c). There is an approximately linear increase relationship between the response and NO_2_ concentration for pure ZnO, Ag/ZnO-10 and Ag/ZnO-20 samples. Specifically, the gas sensing performance increases with increasing photoreduction time of Ag ions from 10 to 30 min. However, further increasing photoreduction time to 40 min induces excessive Ag-NPs, leading to the decrease of gas sensing performance. The influence of Ag-NPs on the gas response performance of Ag/ZnO samples is attribute to the concurrence of two parallel phenomena. 1) Ag-NPs promote oxygen adsorption/dissociation, which consists in catalytic activation of oxygen dissociation on the semiconductor to enhance the surface reactivity. 2) The electronic effect consisting in electron of Ag injected into the C.B. of ZnO can be traced back to the formation of Schottky junctions at the Ag-ZnO interface. These studies hint the potential application of gas sensor based on Ag/ZnO-NAs in monitoring NO_2_.

In summary, aligned h-Ag/ZnO-NAs of nanoparticles-on-nanorods-on-nanowires were fabricated by a multiscale synthesis method via a MES-Writing technology, together with a subsequent hydrothermal growth and photoreduction. Such aligned h-Ag/ZnO-NAs exhibit significant enhancement in gas sensing performance, and this is ascribed to the higher specific area, surface reactivity, and the Schottky junctions at the interface between Ag and ZnO. The fabrication processes shows great influence on the sensing performance as well. And the proposed synthesis method can be used to prepare aligned h-Ag/ZnO-NAs in a digital, large-area and cost-effective manner, which would be a valuable technique for the fabrication of advanced sensing devices.

## Materials and Methods

### Materials

PEO (Polyethylene oxide) is purchased from Aldrich. Zn(CH_3_COO)_2_·2H_2_O (ZnAc), Zn(NO_3_)_2_·6H_2_O, hexamethylenetetramine (HMTA) and silver nitrate(AgNO_3_) are purchased from Sinopharm Chemical Reagent Co., Ltd.

### Synthesis of patterned ZnO nanorod arrays

The aligned nanowires with tunable diameter and distance were directly deposited on substrate via MES-Writing. ZnO-NAs grew on the nanowires by a selective hydrothermal synthesis method. In a typical procedure, an aqueous solution of PEO (600,000, 6 wt%) and ZnAc (0.05 M) was printed by MES-Writing on alumina substrate with Ag interdigital electrodes (IDT). Then the substrate was sintered at 200 °C for 2 hours (h), and a ZnAc seed layer was formed. The seeded substrate was immersed into a Teflon-lined stainless steel autoclave filled with mixed aqueous solution containing 0.05 M Zn(NO_3_) and 0.05 M HMTA. ZnO-NAs grow on nanowires at 90 °C. After 12 h of hydrothermal reaction, the substrate was taken out of the mixed aqueous solution, washed with deionized water, then dried at 60 °C.

### Synthesis of Ag/ZnO-NAs

The Ag/ZnO-NAs were prepared by photodeposition method. The distribution and density of ZnO-NAs with 20 µm distance between nanowires were suitable for Ag-NPs deposition and gas sensitive reaction. AgNO_3_ was dissolved in the mixed solvent of deionized water and absolute ethyl alcohol to form 0.01 M AgNO_3_ solution by magnetic stirring for 0.5 h in dark. The ratio of deionized water to absolute ethyl alcohol was 4:1. The ZnO-NAs were preirradiated with 400 W UV light (wavelength: 365 nm) for 1 h to enhance their hydrophility and then were immersed into 100 mL 0.01 M AgNO_3_ solution in a quartz flask for 1 h to make sure that Ag^+^ ions adsorbed onto ZnO surfaces completely. The above ZnO-NAs were put into another 0.01 M AgNO_3_ solution in a closed container which was filled with flowing pure N_2_ to prevent oxidation. Ag^+^ ions were photoreduced by irradiation of the sample and AgNO_3_ solution with UV light. The Ag-NPs were photodeposited onto the surfaces of ZnO nanorods. The Ag content is controlled through the UV irradiation time. Finally, these samples were rinsed with deionized water to remove the residual Ag^+^. The energy of photon (*hν*) of UV light (3.397 eV) is larger than the band gap of ZnO (*Eg* = 3.37 eV), leading to the generation of electron-hole pairs. Ethanol acts as the hole scavenger to consume the photo-induced holes, leaving the unpaired electrons on ZnO surface^43^. The photogenerated holes (h^+^) in the valence band are consumed to oxidize C_2_H_5_OH to produce ethoxy radicals C_2_H_4_OH**•**. Meanwhile the accumulated electrons (e^−^) in the C.B. contribute to reduce Ag^+^ to form Ag-NPs *in situ* on ZnO nanorods.

### Characterization and Measurement of Gas Sensor

The microstructure of pure ZnO and Ag/ZnO-NAs were characterized by TEM (JEM-2100F STEM/EDXS, JEOL) and FESEM (Sirion200, FEI). The morphologies and crystal planes of the samples were investigated by FESEM at 20 kV sputtering a thin film of Pt and TEM including high-resolution transmission electron microscope (HRTEM) and High-angle annular dark field scanning transmission electron microscopy (HAADF-STEM). The crystalline structure, nanorod growth direction and composition of the samples were analyzed by an XRD (Philips X’Pert PRO diffractometer) with Cu-Kα radiation (*λ* = l.5418 Å). The gas responses of ZnO sensors were measured using a homemade test system. The gas dilution system is used for gas sensing measurement. A resistive heater is utilized for the control of operating temperature. The gas sensors are fixed in the chamber, and Ag interdigital electrodes connect with two probes. 4.096 V (DC) power is supplied to the signal resistor *R*_*0*_ and sensor. The voltage across *R*_*0*_ is continuously recorded as *V*_*0*_, when a ZnO sample is alternately exposure to air and target gas atmosphere at a certain temperature. The resistance of the sensor *R* can be calculated obtained by Ohm’s law.

## Electronic supplementary material

Supplementary information
