# Supplementary material for: Aligned hierarchical Ag/ZnO nano-heterostructure arrays via electrohydrodynamic nanowire template for enhanced gas-sensing properties
Source: Sci Rep. 2017 Sep 22;7:12206. doi: 10.1038/s41598-017-12553-7 (PMC5610263; doi:10.1038/s41598-017-12553-7)
Supplement: Supplementary file 1 — Supplementary information [file 41598_2017_12553_MOESM1_ESM.doc]

Supplementary information:

Aligned hierarchical Ag/ZnO nano-heterostructure arrays via electrohydrodynamic nanowire template for enhanced gas-sensing properties

Zhouping Yin ^1,2,#^, Xiaomei Wang^3, #^ , Fazhe Sun^4^, Xiaohu Tong^5^, Chen Zhu^1,2^, Qiying Lv^6^, Dong Ye^1,2^, Shuai Wang^6^, Wei Luo^5^, and YongAn Huang^1,2,*^

1. State Key Laboratory of Digital Manufacturing Equipment and Technology, Huazhong University of Science and Technology, Wuhan 430074, China
2. Flexible Electronics Research Center, Huazhong University of Science and Technology, Wuhan 430074, China
3. School of Science, Shandong University of Technology, Zibo 255100, China.
4. Analysis Testing Center, Shandong University of Technology, Zibo 255100, China.
5. School of Optical and Electronic Information, Huazhong University of Science and Technology, Wuhan 430074, China
6. School of Chemistry and Chemical Engineering, Huazhong University of Science and Technology, Wuhan 430074, China

# Contributed equally to the paper

* Corresponding author: yahuang@hust.edu.cn

**Sensing mechanism**

It is known that the carrier concentration and carrier mobility determine the conductivity of ZnO. The main carriers are free electrons of the conduction band (C.B.) for n-type ZnO. The n-type donors in ZnO semiconductor are oxygen vacancies which are shallow states. The C.B. of ZnO can accept the donated electrons from the adjacent cations whose bonding electrons is easily removed. The concentration of free electron produced by oxygen vacancies is high. Atoms and molecules are tend to bind at the oxygen vacancy sites on semiconductor surface[^1^](#_ENREF_1). The main charge acceptor on ZnO surface are oxygen molecule. Thus the free electrons in C.B. of ZnO ionized the adsorbed oxygen species, and ionized oxygen species such as O_2_^−^, O^−^ and O^2−^were formed[^2^](#_ENREF_2). Oxygen adsorption reactions highly depend on working temperature, and the stable oxygen ions are O_2_^−^, O^−^ and O^2−^ at below 100 ^◦^C, within 100-300 ^◦^C and above 300 ^◦^C operating temperature, respectively[^3^](#_ENREF_3). The lower concentration of free electrons in C.B. of ZnO made the increase of resistance[^4^](#_ENREF_4). A balance turn out between the free electrons in ZnO nanomaterial and the captured electrons on the surface, and the balance changes when ZnO sample is put into target gas. The resistance of ZnO samples is modulated by the adsorption and desorption of gas molecules.

Upon exposure to NO_2_ gas, NO_2_ gas molecules are adsorbed on the surface of ZnO nanorods. NO_2_, as an oxidizing gas and electron acceptor, attracts the free electrons from the C.B. of ZnO. The reactions are as follow:

$$NO_{2}(g)+e^{-}\to NO_{2}^{-}(a)$$

$$NO_{2}(g)+e^{-}\to NO(g)+O^{-}$$

where (g) and (a) mean “gas” and “adsorbed”, respectively, representing the existing state of molecules or ions. NO_2_ molecules have higher electrophilic property, and they can not only capture the electronics from ZnO, but also react with the oxygen ions, the reactions can be described as follows[^5^](#_ENREF_5):

$$NO_{2}^{-}(a)+O_{2}^{-}(a)+e^{-}\to NO_{2}^{-}(a)+2O^{-}(a)$$

$$NO_{2}^{-}(a)+2O^{-}(a)+e^{-}\to NO_{2}(g)+2O^{2-}(a)$$

NO_2_ and $NO_{2}^{-}$ have good surface reaction stabilities at 225 ^◦^C operating temperature[^6^](#_ENREF_6). The electron concentration decreases through these series of reactions, and the surface depletion region of ZnO nanorod is further widen, so the resistance of the sensor further increase [^7^](#_ENREF_7). Thus, the cycling reaction continued:

$$NO_{2}(g)+e^{-}\to NO_{2}^{-}(a)$$

When NO_2_ concentration increases, more electrons will be involved in above reactions, and the depletion layer width increase. As a result, the resistance of the sensor increases with NO_2_ concentration increased.

**References**

1 Feng, P., Shao, F., Shi, Y. & Wan, Q. Gas Sensors Based on Semiconducting Nanowire Field-Effect Transistors. *Sensors* **14**, 17406 (2014)

2 Şahin, Y. *et al.* Electrical conduction and NO_2_ gas sensing properties of ZnO nanorods. *Appl. Surf. Sci.* **303**, 90-96 (2014)

3 Ghimbeu, C. M., Schoonman, J., Lumbreras, M. & Siadat, M. Electrostatic spray deposited zinc oxide films for gas sensor applications. *Appl. Surf. Sci.* **253**, 7483-7489 (2007)

4 Shi, L. *et al.* Highly Sensitive ZnO Nanorod- and Nanoprism-Based NO_2_ Gas Sensors: Size and Shape Control Using a Continuous Hydrothermal Pilot Plant. *Langmuir* **29**, 10603-10609 (2013)

5 Bai, S. L. *et al.* Gas Sensing Properties of Quantum-Sized ZnO Nanoparticles for NO_2_. *IEEE Sens. J.* **12**, 1234-1238 (2012)

6 Rai, P., Kim, Y.-S., Song, H.-M., Song, M.-K. & Yu, Y.-T. The role of gold catalyst on the sensing behavior of ZnO nanorods for CO and NO_2_ gases. *Sens. Actuators, B* **165**, 133-142 (2012)

7 Park, S., An, S., Mun, Y. & Lee, C. UV-Enhanced NO_2_ Gas Sensing Properties of SnO_2_-Core/ZnO-Shell Nanowires at Room Temperature. *ACS Appl. Mat. Interfaces* **5**, 4285-4292 (2013)
